# Facile Ball Milling Preparation of Flame-Retardant Polymer Materials: An Overview

**DOI:** 10.3390/molecules28135090

**Published:** 2023-06-29

**Authors:** Xiaming Feng, Xiang Lin, Kaiwen Deng, Hongyu Yang, Cheng Yan

**Affiliations:** 1 College of Materials Science and Engineering, Chongqing University, 174 Shazhengjie, Shapingba, Chongqing 400044, China; 2 Department of Mechanical Engineering, Southern University and A&M College, Baton Rouge, LA 70813, USA

**Keywords:** ball milling, flame retardants, polymer composites, exfoliation, fire retardancy

## Abstract

To meet the growing needs of public safety and sustainable development, it is highly desirable to develop flame-retardant polymer materials using a facile and low-cost method. Although conventional solution chemical synthesis has proven to be an efficient way of developing flame retardants, it often requires organic solvents and a complicated separation process. In this review, we summarize the progress made in utilizing simple ball milling (an important type of mechanochemical approach) to fabricate flame retardants and flame-retardant polymer composites. To elaborate, we first present a basic introduction to ball milling, and its crushing, exfoliating, modifying, and reacting actions, as used in the development of high-performance flame retardants. Then, we report the mixing action of ball milling, as used in the preparation of flame-retardant polymer composites, especially in the formation of multifunctional segregated structures. Hopefully, this review will provide a reference for the study of developing flame-retardant polymer materials in a facile and feasible way.

## 1. Introduction

Polymer materials have contributed significantly to the development of modern society, due to their excellent properties, including high workability, low price, and good chemical resistance [1,2,3,4,5,6]. However, the flammability of most polymer materials greatly limits their wide range of applications [7,8,9,10]. One proposed solution to this problem is the development of flame retardants [11,12,13,14], which could restrain the ignition and fire-spreading of polymer materials, specifically decreasing heat release and smoke production. To date, various flame retardants have been developed to achieve satisfactory fire resistance in different polymer materials, such as halogenated flame retardants [15,16,17,18,19], inorganic layered compounds [20,21,22,23], and phosphorous–nitrogen intumescent flame retardants [24,25,26,27]. Flame-retardant polymers and their composites have been used widely in construction, electronics, transportation, and so on [28,29,30]. Regarding the preparation of flame retardants, the conventional method is liquid-phase synthesis [31,32], in which organic solvents and complicated purification are always required. Moreover, the environmental toxicity and environmental accumulation of various flame retardants have become increasingly important [33,34,35]. Therefore, for high efficiency and environmental protection, a more efficient and greener approach toward the facile preparation of flame retardants is highly desirable.

As a typical sub-factor in mechanochemistry [36], ball milling has been developed for crushing, mixing, and reacting, due to the impact and shear forces generated by high-speed rotation and high-temperature surroundings [37,38,39,40]. It is widely used in fabricating flame retardants, for its superior properties of easy processing, low cost, and large-scale production [39]. The most conventional application of ball milling in the field of flame retardants is grinding to reduce particle size [41,42]. Notably, ball milling is effective in preparing inorganic compounds on the nanoscale [43,44,45], including flame-retardant synergists. As is well known, the size of additives for polymer composites is closely related to the dispersion state and the final performance. Recently, with the rise of two-dimensional nanomaterials (e.g., graphene), the shear force generated by ball milling is utilized to achieve the facile exfoliation of layered compounds, such as graphite [46,47,48], boron nitride [49,50,51,52], and black phosphorus [53,54]. Another important usage of ball milling is to conduct chemical reactions, including the simple surface modification of particles, and complicated chemical synthesis [14,55]. As for flame retardants, surface modification by ball milling is prominent in improving properties, such as hydrolysis resistance [56,57], interfacial compatibility [56,57], and flame-retardant efficiency [58,59]. Moreover, ball milling plays an important role in fabricating flame-retardant polymer composites, including simple mixing, and customizing specific structures (e.g., a segregated structure for electromagnetic wave shielding) [60,61].

This review aims to summarize the technological progress made in utilizing ball milling for facilely preparing flame retardants, followed by a study and discussion of the fabrication of flame-retardant polymer composites. Firstly, the basic concept and the category of ball milling are briefly discussed. Next, the crushing, exfoliating, modifying, and reacting actions of ball milling in developing flame retardants are primarily examined. Then, ball milling for the mixing of flame retardants and the polymer matrix, which concern segregated structures and flame retardancy, is overviewed. Finally, conclusions are proposed, and insights are given.

## 2. Ball Milling Methods

Ball milling is a technique that is widely utilized to crush powders into small particles. According to the operational mode, the types of ball milling primarily include planetary ball milling, tumbler ball milling, vibration ball milling, and attrition ball milling. Each type of mill is developed to achieve a specific purpose, and each undoubtedly has relative weaknesses. For example, attrition ball milling generates higher surface contact, while vibration ball milling could produce higher milling force [62,63]. Comparatively, planetary ball milling is widely used owing to its compact size and low cost. According to the generated energy, ball milling can be mainly classified as low-energy ball milling and high-energy ball milling. The rotation speed of most planetary ball mills is 0~500 rpm, while high-energy ball mills can reach up to 1800 rpm. During ball milling, shear force, impact force, and friction force can be generated. Shear force is in favor of exfoliating layered compounds, and impact force can efficiently grind the powders into fine particles. As for high-energy ball milling, the heat generated by friction can be used to induce the chemical reaction and phase transition, such as the conversion of red phosphorus to black phosphorus. Regarding the materials of the ball milling tank and bead, the most commonly used is made from steel, due to the high hardness and processability. Other widely used materials include agate, zirconia, and nylon, which are appropriate for those raw materials that can react with steel. That is to say, the ball milling method satisfies almost all requirements when developing flame retardants, including crushing, exfoliating, surface modifying, and reacting, as well as mixing flame-retardant additives and the polymer matrix without the limitation of containers.

## 3. Ball Milling-Assisted Fabrication of Flame Retardants

### 3.1. Ball Milling for Crushing

**Figure 1:**
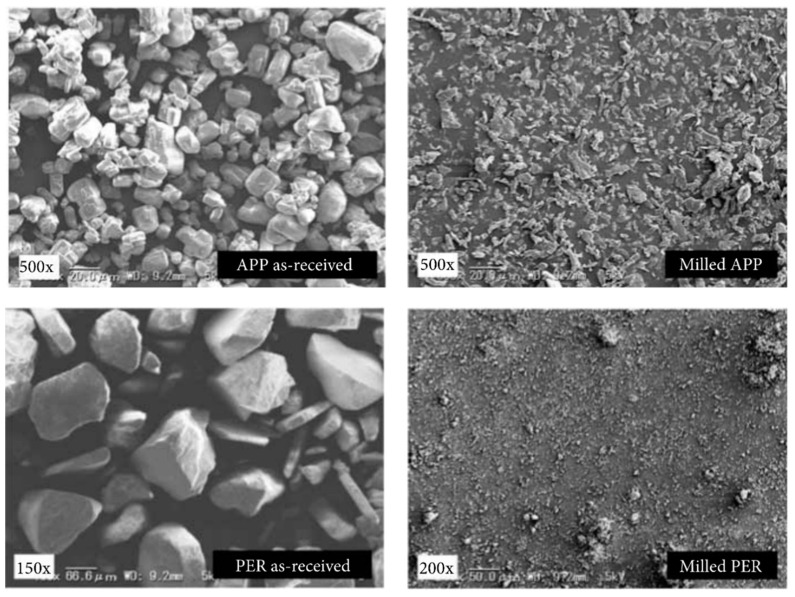
SEM micrographs of APP and PER additives before and after milling. Reproduced from ref. [64] with permission from John Wiley and Sons.

The basic application of ball milling is crushing powders, including flame-retardant additives. As is known, the particle size plays an important role in influencing the properties. For example, the specific surface area increases with the reduction in particle size, which is crucial for flame retardants with catalytic effects. As for mechanical properties, large flame-retardant particles lead to a stress concentration within polymer composites. As a result, the mechanical strength is always decreased. Therefore, crushing flame retardants into fine powders is necessary. Bocz and coworkers studied the influence of flame retardant size on the fire retardancy and mechanical properties of polypropylene (PP) composites [64]. The flame-retardant system consists of pentaerythritol (PER) and ammonium polyphosphate (APP) in a weight ratio of 1 to 2. Ball milling was utilized to reduce the particle size of the APP/PER mixture to a large extent, as shown in Figure 1. Upon undergoing the ball milling process, the average particle size of APP was decreased from 15 µm to 8 µm, while the PER was crushed from microparticles (~200 µm) to submicronic particles. An outperformed flame retardancy was observed in the PP composite with smaller APP/PER particles. It is believed that the better distribution of additives and the modified degradation mechanism contribute significantly to the formation of a protective char layer. Moreover, the smaller APP/PER particles are in favor of enhancing the mechanical performance (10% higher tensile strength) of flame-retarded PP composites.

In Bao’s research, the attapulgite clay (ATP) was treated by ball milling to reduce the particle size. It was then grafted onto cotton fabric to improve flame retardancy [65]. To comprehensively evaluate the effect of the milling process, the ball milling was conducted for 4, 5, 6, and 8 h, under rotational speeds of 200, 300, and 400 r/min, in the mass ratio of zirconia ball to ATP of 3:1, 4:1, 5:1, and 6:1, respectively. It was observed that the ball milling parameters (speed, time, and mass ratio) strongly influenced the particle size of the milled ATP, as well as the final fire retardancy of the resultant cotton fabrics. The uniformly distributed stable oxide layer decomposed by the ATP was believed to be the key contributor. Following the same idea, Üreyen and partners crushed zinc borate (ZnB) from 9 μm to a submicron scale by wet milling, and subsequent high-shear-fluid processing, to reduce the flammability of polyethylene terephthalate (PET) woven fabrics [66]. The crushed ZnB was dispersed in alkyl phosphonate and organophosphorus flame retardants, to obtain a homogenous dispersion, which was then applied to the fabrics by the pad-dry-cure method. A synergistic effect between ZnB and organophosphorus flame retardants was proposed, specifically decreasing the mean CO, total smoke release, and total smoke production.

In recent years, the concept of sustainable development has been defined as essential to combating climate change. The combination of facile preparation and biomass raw materials attracts much attention in developing flame retardants. Among these, the ball milling of various biomass materials is of great importance. For example, Jawaid and coworkers reported the preparation of nano flame retardants from date palm biomass [67]. They performed the agricultural waste, date palm trunk fiber as biobased raw material upon the chemical process, and high-energy ball milling to fabricate new flame retardants on the nanoscale. The particle size analysis results indicated that the as-prepared products were in the mean size range of 274.5–289.7 nm. The multielement composition (carbon, oxygen, silicon, sulfur, calcium, and potassium) and high decomposition temperature of nano-sized fillers suggest their potential application in the field of flame retardants [68,69]. Besides the biomass raw materials, another important issue in consideration of critical environmental pollution is how to reuse the existing petroleum-based material [70,71,72]. Wang and coauthors proposed a novel recycling strategy for fabricating fire retardants from polyphenylene sulfide waste textiles [73]. A sequence of thermal aging, ball milling, and screening was conducted to upcycle waste polyphenylene sulfide (PPS) filter bags into PPS powders (75–100 μm), which were first used to decrease the flammability of epoxy resin (EP). The high thermal stability and good charring ability of PPS are believed to constitute the main reason for the decreased heat and smoke/toxic gases released from EP composites. This work presented a promising pattern in the upcycling of solid wastes into flame retardants.

### 3.2. Ball Milling for Exfoliation

As a typical type of inorganic flame-retardant additive with a physical barrier effect, nano-sized layered compounds have been a hotspot for flame-retardant research in recent years, from clay to graphene to MXene. The preparation of nano-sized layered flame retardants generally includes the bottom-up approach and top-down processing. Comparatively, the top-down approach features the superiority of easy processing, large-scale production, and low cost. Owing to the shear forces, among other aspects, ball milling is considered one of the most promising techniques for exfoliating layered compounds and producing two-dimensional flame retardants.

#### 3.2.1. Graphene-Based Flame Retardants

**Figure 2:**
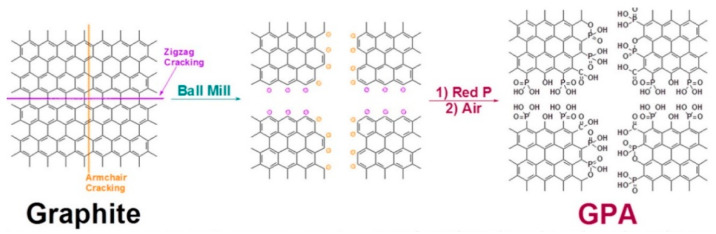
Schematic representation of the mechanochemical cracking of a graphite flake in a ball-mill crusher containing stainless steel balls (diameter 5 mm), in the presence of red phosphorus, and subsequent exposure to air moisture to produce GPA. Reproduced from ref. [46] with permission from the American Chemical Society.

The discovery of graphene has opened up a whole new field of material research. Graphene has been regarded as a promising material in various fields, including electronics [74,75], catalysts [76,77], and semiconductors [78,79], as well as flame retardants [80,81]. However, the high cost of preparing graphene is always a critical problem, especially in the flame-retardant field, which sees such high consumption. Researchers have developed various methods for fabricating graphene on an acceptable production scale. Ball milling is always a good choice. Kim and coworkers initially reported the fabrication of graphene phosphonic acid (GPA) flame retardants via the ball milling of graphite and red phosphorus [46]. As shown in Figure 2, the graphite is firstly crushed and exfoliated into thin layer graphene. Upon exposure to the high energy generated by ball milling, the graphitic C-C bonds cleave, and react with phosphorus to form C-P bonds. Subsequently, the unstable P converts into a phospho-oxygen compound, and then phosphate compounds (highest oxidation state), in the presence of oxygen and moisture. The obtained GPA can be easily dispersed in various solvents to form a stable solution, which could be used to treat papers and fabrics, to allow fire retardancy.

Inspired by Kim’s work, Jeon and coworkers prepared a heavily aluminated graphene (AlGnP) flame retardant through the ball milling of graphite and solid aluminum (Al) beads. Approximately 30.9 wt% of element Al in AlGnP was detected [47]. Subsequently, the poly(vinyl alcohol)/AlGnP composite films were fabricated via a simple solution blending and casting method, thanks to the excellent dispersibility of AlGnP. As expected, an improved flame retardancy was observed. Additionally, Chen and coworkers prepared Sn-doped graphene (GnPSn) as an efficient flame retardant, via ball milling expandable graphite and Sn powder in a wet condition [48]. The synergistic flame-retardant effect of GnPSn and hexaphenoxy cyclotriphosphazene (HPCTP) for EP resin was proposed. An LOI value of 33.6%, and UL-94 of V0 grade, were observed for the EP composite with 6.3 wt% HPCTP and 2.7 wt% GnPSn. The compactness of the residual char in the condensed phase was highlighted in discussing the specific flame-retardant mechanism.

For the efficient exfoliation of graphite, a combination of ball milling and other techniques has been proposed, such as ball milling coupled with ultrasonication, reported by Wang et al. [82], thermal shock combined with the ball milling method [83], and a combination of the ball milling and microwave-assisted methods [84]. Moreover, to meet the goal of sustainable development, various green and biobased materials have been applied to assist the ball milling-induced exfoliation of graphite, such as waste fish deoxyribonucleic acid and Acacia mangium tannin (AMT) [80,85]. As expected, the green exfoliating agent can not only enhance the exfoliation efficiency of graphite, but can also improve the dispersibility and flame-retardant capability.

#### 3.2.2. Boron-Nitride-Based Flame Retardants

Boron nitride is another important layered compound, which is highly thermally conductive, but electrically insulated [51]. Different from graphene, boron nitride nanosheets are widely used to fabricate polymer nanocomposites with high thermal conductivity, while maintaining electric insulation properties, which are promising in the field of thermal management with high fire risk [50,52]. Certainly, boron nitride nanosheets can be an efficient type of flame retardant, due to their high thermal stability and specific nano-size effect. Based on the same exfoliation mechanism, ball milling is often used to achieve the exfoliation of bulk boron nitride. Qiu and coworkers realized the scalable production of hydroxyl-functionalized BN (OBN) by simple ball milling and annealing under air conditions, in the presence of sodium hydroxide [49]. The shear-force-induced exfoliating and chemical peeling were believed to be the key points. The resultant OBN could be a platform to load and graft various flame-retardant units, to improve the fire safety of EP resin. As a result, the EP composites exhibited not only enhanced fire retardancy, but also an improved friction performance.

Inspiringly, Han et al. included the conventional flame-retardant ammonium phosphate in the above-mentioned ball milling exfoliation process, as an assistant agent [51]. A synergetic action between the shear and chemical peeling of ammonium phosphate and sodium hydroxide was observed. Density functional theory (DFT) calculations were performed, to reveal the possible mechanochemical reaction mechanisms. As expected, the resultant BN nanosheets endowed EP resin with exceptional fire retardancy, including 60.9%, 35.7%, 44.3%, and 38.8% reductions in PHRR, THR, SPR, and TSP, respectively. The catalytic charring effect and physical barrier action of BN were highlighted in the improvement of flame retardancy. Subsequently, the same group reported the fabrication of ionic liquid-wrapped boron nitride nanosheets (BNNS@IL) using the ball milling process [52]. The exfoliation and functionalization were achieved via a similar mechanochemical action. Based on the BNNS@IL, a fire resistant EP-based thermally conductive layered film with aligned BN nanoflakes was developed, which showed high anisotropic thermal conductivity (K_‖_ of 8.3 and K_⊥_ of 0.8 W m^−1^ K^−1^) and excellent flame retardancy, suggesting new possibilities in electrical device and thermal management. Additionally, the nitrogen-phosphorus-doped boron nitride (BN@APP) was prepared by Xu and coworkers via the ball milling method [86]. The effect of BN@APP on the flame retardancy and thermal conductivity of polybutylene succinate (PBS) was comprehensively studied. A 62.8% increase in thermal conductivity, and a 44.8% decrease in TSP, were reported.

**Figure 3:**
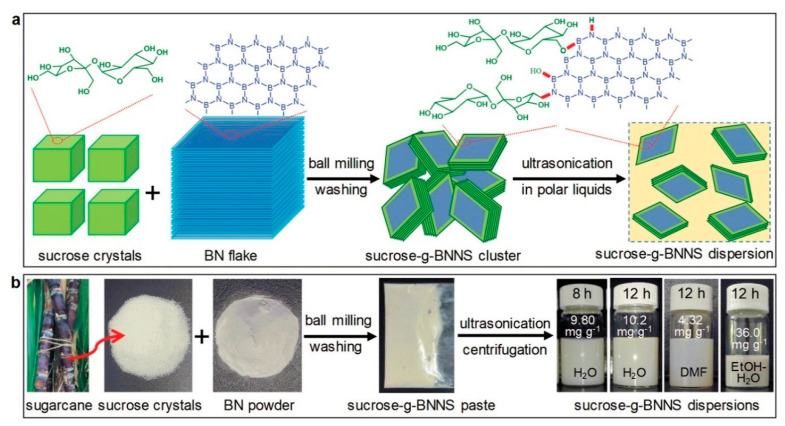
(**a**) Scheme of the exfoliation procedure. (**b**) Photos of the raw materials, the sucrose-g-BNNS paste, and sucrose-g-BNNS dispersions in H_2_O, DMF, and ethanol (EtOH)-H_2_O mixture [50]. Reproduced with permission from John Wiley and Sons.

To improve the exfoliation efficiency of boron nitride, a sugar-assisted mechanochemical exfoliation (SAMCE) method was developed by Chen’s group [50]. As shown in Figure 3, the sugar (sucrose) molecules can be covalently grafted to BN nanosheets during ball milling, which efficiently prevents restacking, and leads to the high exfoliation yield of 87.3%. The obtained BN can be uniformly dispersed in water and organic solvents, due to the grafted sucrose molecules. As a result, the exfoliated BN can greatly reinforce the flexible and transparent poly(vinyl alcohol) (PVA) film, in terms of improved tensile strength, thermal dissipation capability, and fire retardancy. It is believed that this SAMCE method can be extended to the exfoliation of other layered materials, such as black phosphorus.

#### 3.2.3. Black-Phosphorus-Based Flame Retardants

**Figure 4:**
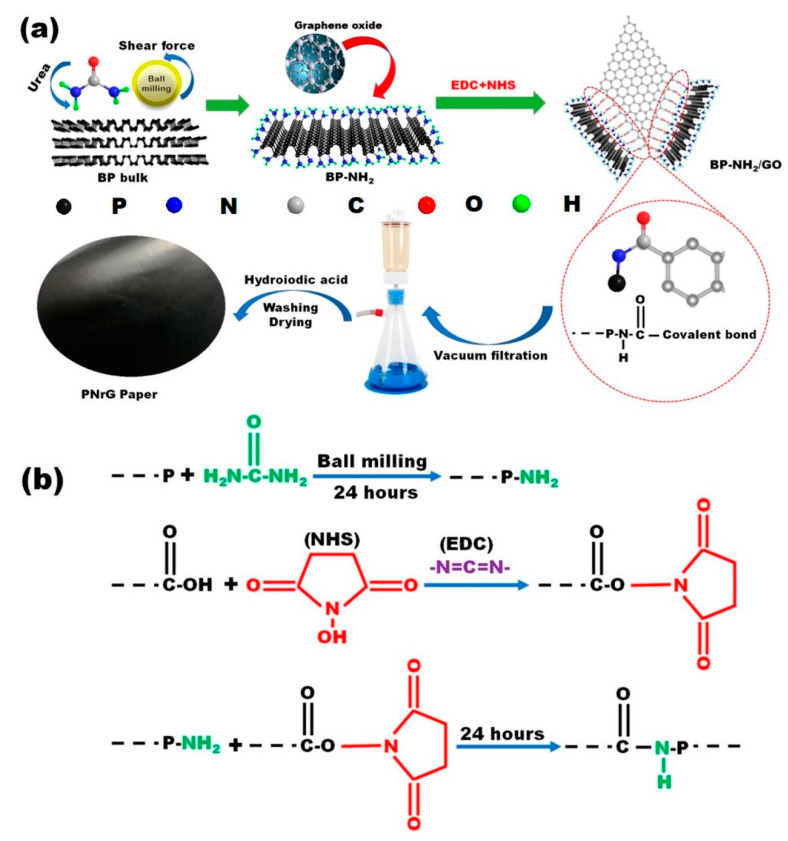
(**a**) Fabrication process of the RPNG film; (**b**) the mechanism of the covalent bond connection between BP-NH_2_ and GO [91]. Reproduced with permission from the American Chemical Society.

Elementary phosphorus is an efficient flame retardant, including red phosphorus [87,88] and black phosphorus [89,90]. Compared to amorphous red phosphorus, layered black phosphorus integrates not only the gas phase and condensed phase flame-retardant mechanism, but also the physical barrier effect, similar to graphene, which has drawn much attention in recent decades. Similarly, ball milling can be used to exfoliate black phosphorus into few-layer nanosheets called phosphorene [53]. The difference is that black phosphorus nanosheets are not stable in open air, and can rapidly degrade into phosphate compounds upon oxidation and hydrolysis. Therefore, for black phosphorus, exfoliation and subsequent protection are of equal importance. Qu and coworkers reported the preparation of aminated black phosphorene (BP-NH_2_) via the ball milling method [91], as shown in Figure 4. The graphene oxide (GO) was covalently bonded to black phosphorus through the reaction of -NH_2_ and -COOH in the presence of a catalyst. The obtained product was assembled into a flexible film (RPNG) with ultrahigh thermal conductivity and remarkable flame retardancy, which exhibited a fantastic application in fire alarm sensors. Along the same lines, they bonded the multi-walled carbon nanotubes (MWCNTs) to black phosphorus nanosheets via the -NH-CO- linkage [92]. The resultant nanofiller (BP-MWCNTs) could endow cellulose nanofiber (CNF) with satisfactory thermal conductivity and fire retardancy, specifically an in-plane thermal conductivity of 22.38 ± 0.39 W m^−1^ K^−1^, and a cross-plane thermal conductivity of 0.36 ± 0.03 W m^−1^ K^−1^, UL-94 V-0 grade, and a LOI value of 29.9%.

Guo and coworkers reported the simultaneous exfoliation and functionalization of black phosphorus via sucrose-assisted ball milling, with N-methyl pyrrolidone (NMP) intercalating for high efficiency [53]. They found that the sucrose molecules could protect black phosphorus from oxidating, and promote the dispersion of black phosphorus nanosheets in solvents. The sucrose-grafted BP dramatically enhanced the mechanical performance and flame-retardant property of PVA, in terms of a 131.2% increase in tensile strength, and a 52.5% reduction in PHRR. The encapsulation effect of sucrose was highlighted in the exceptional air stability of PVA nanocomposite films. In Duan’s work, ball milling, liquid exfoliation, and electrochemical exfoliation were applied to prepare black phosphorus nanosheets with different sizes, to clarify the size-dependent flame retardancy of black phosphorus nanosheets [54]. EP resin was selected as the polymer matrix. They found that the liquid ball milled BP (lb-BP) was the best at dispersing in the EP matrix, and endowed the EP with the highest flame retardancy. The barrier and carbonization catalyst action of lb-BP was believed to be the primary cause of the delayed combustion.

#### 3.2.4. MoS_2_-Based Flame Retardants

**Figure 5:**
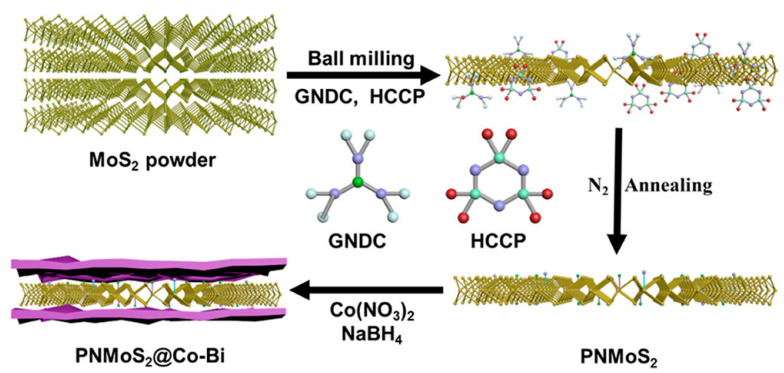
Synthetic route of the nanostructure PNMoS_2_@Co–Bi [93]. Reproduced with permission from the American Chemical Society.

Molybdenum disulfide (MoS_2_) consists of the elements Mo and S. Mo is a transition metal and demonstrates catalytic ability and smoke suppression performance [93,94]; S is also a flame-retardant element. MoS_2_ has a layered structure, and can be exfoliated into nanosheets, the high specific area of which is in favor of improving catalytic performance. Therefore, the exfoliation of MoS_2_ for developing high-performance flame retardant is highly desirable. Qiu and coworkers exfoliated MoS_2_ into nanolayers via a ball milling method. Subsequently, a high-temperature polymerization was conducted to obtain the polyphosphazene nanoparticle (PPN) functionalized MoS_2_ nanosheets (MoS_2_@PPN) [95]. It was revealed that the loaded PPN could prevent the restacking of MoS_2_ nanolayers, and improve the flame-retardant capability. Upon the incorporation of MoS_2_@PPN, the flame retardancy and friction properties of the EP composite were improved. Based on the ball milling-exfoliated MoS_2_ nanosheets, Zou’s group constructed a phosphorus/nitrogen-co-doped MoS_2_/cobalt borate nanostructure as a flame-retardant and anti-wear additive [93], as displayed in Figure 5. The GNDC and HCCP were used to assist in the exfoliation and modification of the MoS_2_. After annealing, a two-dimensional cobalt borate (Co−Bi) nanosheet could be generated onto the MoS_2_ nanosheets, resulting in a novel MoS_2_-based flame retardant (PNMoS_2_@Co−Bi). With only 2 wt% addition of PNMoS_2_@Co−Bi, the EP composite exhibited a much-decreased flammability, and the detailed mechanism was clarified.

#### 3.2.5. Covalent-Organic-Framework-Based Flame Retardants

Covalent organic frameworks (COFs) have a layered structure and flame-retardant action, due to their unique elementary composition [96,97]. Mu and coworkers explored the exfoliation of COFs by ball milling, and demonstrated their flame-retardant performance on thermoplastic polyurethanes (TPU) and polypropylene (PP) [98]. Firstly, they proposed the preparation of novel melamine/o-phthalaldehyde COF nanolayers. Details of the preparation, exfoliation, dispersion state, and flame-retardant performance of melamine/o-phthalaldehyde COFs were discussed. Then, to improve flame-retardant ability and smoke suppression, the Co_3_O_4_/COF nanohybrids were prepared based on the ball milling-induced exfoliation of the COFs [99]. The fire retardancy, smoke and carbon monoxide (CO) suppression, and thermal stability of the PP composites were characterized. A synergistic effect between Co_3_O_4_ and COFs was concluded.

#### 3.2.6. Layered Oyster Waste

**Figure 6:**
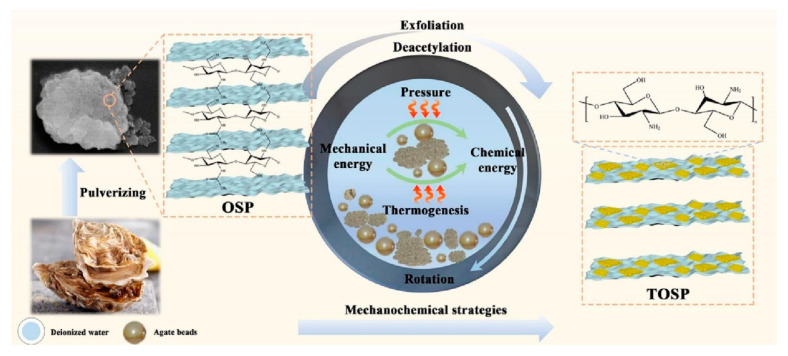
The preparation procedure for the TOSP [100]. Reproduced with permission from Elsevier.

Most recently, Chen and his group have focused their attention to recycling layered oyster wastes as flame retardants. The layered oyster consisted of 95% layered CaCO_3_, and 5% organic adhesives. However, the flame-retardant action of the bare oyster waste powders was unsatisfactory. Therefore, proper processing was highly desired in order to achieve the deconstruction and modification of layered oyster waste [100]. Firstly, they applied a simple ball milling of oyster powders, to obtain a phosphorus-free hybrid flame-retardant (TOSP), as shown in Figure 6. The successful exfoliation of layered oyster waste was confirmed. Moreover, the flame retardancy of the EP composite with the addition of milled layered oysters was investigated. This work opened a concept-new way to upcycle oyster waste, as high-value flame retardant, with the assistance of ball milling. To improve the flame-retardant ability of oyster wastes, the same group successively used ammonia phytate (PAA) [101] and chitosan-modified ammonium polyphosphate (CS@APP) [102] in assisting the ball milling-induced exfoliation of layered oyster wastes. This revealed that the resultant TOSP@PAA and TOSP@CS@APP were of a specific layer-crosslinking structure, by the –NH^3+^–O^–^P– bonds. As a result, the flame-retardant actions of TOSP@PAA for EP, and TOSP@CS@APP for cotton fabric, were higher than the common TOSP.

#### 3.2.7. Others

Besides the above-mentioned layered materials, some other layered flame-retardant compounds can be exfoliated by the ball milling method, such as layered double hydroxide (LDH) [103], Mxene [55], flake-NiNH_4_PO_4_·H_2_O (IL-ANP) [104], and kaolin [105]. The primary thinking is the same for the processing of these compounds via the ball milling method, including shearing for exfoliation, and in situ modification for improving dispersibility and flame-retardant performance. For example, Huang’s group ball-milled the LDH and red phosphorus to prepare P-LDH flame retardants for TPU [103]. It is believed that the anion substitution and high exfoliation of the LDH nanosheets greatly contributed to the high performance of the TPU composites. He and his coauthors conducted the exfoliation and functionalization of MXenes in the presence of poly(diallyldimethylammonium chloride) (PDDA) by ball milling, to improve the flame retardancy of polyurethane [55]. Notably, the 3 wt% PDDA-modified MXene could efficiently reduce heat release and smoke production.

### 3.3. Ball Milling for Modification

**Figure 7:**
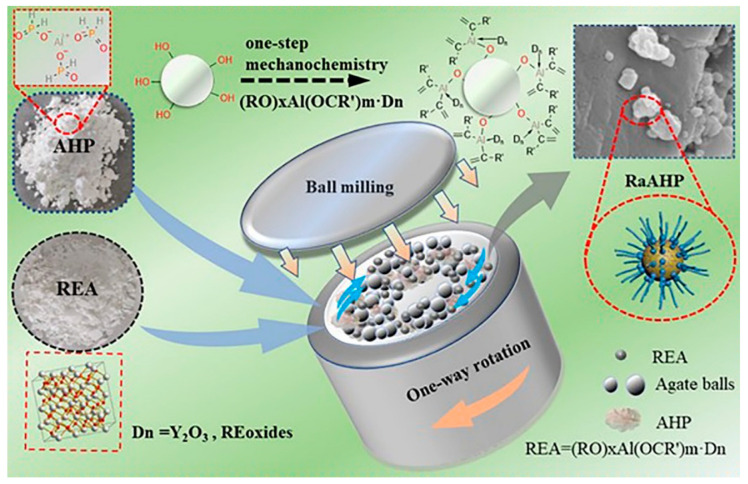
The synthesis process of RaAHP [106]. Reproduced with permission from John Wiley and Sons.

Another important application of ball milling is achieving the surface modification and functionalization of flame retardants for various purposes. For example, to enhance the hydrophobic property of aluminum hypophosphite, a rare earth-based coupling agent (REA) was utilized to modify the aluminum hypophosphite (AHP) through one-step ball milling [106], as shown in Figure 7. This revealed that the AHP modified by 4 wt% REA (RaAHP-4) had an outstanding hydrophobic performance, with a water contact angle of 94.6. Additionally, the EP/6RaAHP-4 composites behaved the best at reducing fire risk, including a decreased heat release and CO production. Guo and coworkers applied the titanate coupling agent NDZ-201, to modify the conventional IFRs composed of melamine (MEL), APP, and pentaerythritol (PER), via ball milling, to enhance thermal stability and dispersity. A cooperative effect on the fire retardancy of the PP composites was clarified. To address the same problem, Yan and coworkers conducted the surface modification using the silane coupling agent KH-550, via wet ball milling [38]. The resultant modified IFRs were used as flame retardants for polyphenylene oxide (PPO). A synergistic effect was observed between PPO and IFR in improving thermal stability and fire retardancy.

Filler additives have drawn much attention in the development of polymer composites, due to the high length-diameter ratio. However, the interfacial compatibility always limits the properties and applications of fiber-reinforced polymer composites. Członka and coworkers modified the walnut shell filler with selected mineral compounds: perlite, montmorillonite, and halloysite, via ball milling [107]. The rheological properties, mechanical properties, thermal properties, and fire retardancy of the fiber-reinforced polyurethane (PUR) composites were comprehensively investigated. A considerable reduction in heat release and smoke production was observed. Similarly, the vermiculite fillers were modified with casein, chitosan, and potato protein, with the assistance of ball milling, to reinforce the flame retardancy of polyurethane foams [108]. Approximately 2 wt% of vermiculite fillers were added. The rheological, thermal, and mechanical properties, and fire resistance, were explored in detail. Additionally, to the same ball milling method, Członka et al. reported the surface modification of lavender fillers with kaolinite and hydroxyapatite, for developing flame-retardant PU composites [109]. Notably, the ball milling-assisted surface modification could be extended to metal oxide flame retardants, such as Sb_2_O_3_ [110] and ZnO [111].

### 3.4. Ball Milling for Reaction

**Figure 8:**
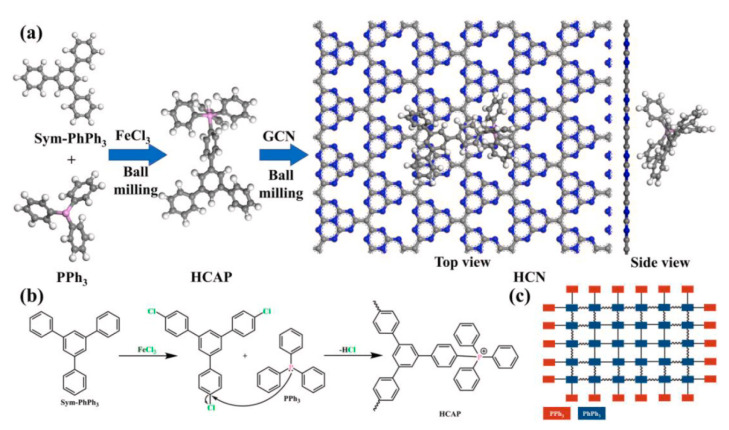
(**a**) Preparation scheme of HCN heterostructure. (**b**,**c**) A possible mechanism of HCAP synthesis [112]. Reproduced with permission from Elsevier.

The heat and force generated by ball milling are sufficient for achieving some chemical reactions, including the solvent-free solid–solid reaction. Although it is not a dominant trend, there are indeed some reports on ball milling in the synthesis of novel flame retardants. Chen and coworkers reported a solvent-free ball milling method of fabricating phosphorus-containing hyper-crosslinked aromatic polymer (HCAP) from triphenylphosphine [112], as shown in Figure 8. Subsequently, nitrogen-rich graphitized carbon nitride was added, to synthesize a series of phosphorus and nitrogen-containing heterojunction flame retardants known as HCN. The molecular structures of HCAP and HCN were well characterized through experimental and computational approaches. The flame-retardant effect of HCN on EP resin was systematically investigated. It was found that the addition of 5 wt% HCN could endow EP resin with a UL-94 V0 rating, and dramatically reduce heat release and smoke production. Moreover, the machine learning method was performed, to evaluate the combined scores of multiple flame-retardant properties. It was believed that charring ability dominated the exceptional flame-retardant effect. This report displayed a brand-new pathway for developing high-performance flame retardants.

How to endow biobased materials (e.g., cellulose crystals) with a flame-retardant effect is a critical problem when considering their high-value application. Apart from the conventional solution method based on corrosive concentrated phosphoric acid, Fiss and coworkers reported a phosphorylation process in cellulose nanocrystals, as well as some polymers, via ball milling [113]. It provided a feasible method to develop various flame retardants based on natural products, such as chitin nanofibril [39] and cellulose nanofibril [114]. Zhang and coworkers successively prepared chitin nanofibril-based flame retardants and cellulose nanofibril-based flame retardants, to improve the fire resistance of papers [39]. The ball milling was conducted in the presence of chitin/cellulose and P_2_O_5_. The degree of phosphorylation was examined in detail using X-ray photoelectron spectroscopy (XPS). As for chitin nanofibril-based flame-retardant-treated papers, an LOI of 30%, and a 62% reduction in PHRR, were obtained, compared to the control paper. Therefore, ball milling has been proven to be an efficient technique in developing novel flame retardants, which is likely to generate further interest.

## 4. Ball Milling for Mixing Flame Retardants and Polymer Matrices

As well as the preparation of flame-retardant additives, ball milling is often used to uniformly mix flame retardants and the polymer matrix, which is, namely, simply blending different particles in solid form. For example, Xu and coworkers conducted the mechanical ball mixing of montmorillonite (MMT), nano-Sb_2_O_3_, BEO, and PP by a high-energy ball milling machine, to fabricate flame-retardant PP composites [115]. To achieve the processing and improve compatibility, Liu and coworkers performed solid-state shear milling for magnesium hydroxide (MH) flame-retardant PP [116]. Upon milling, the pulverization of the PP, high-degree blending, uniform dispersion of MH, and chemical interaction between PP and MH could be obtained at the same time. Compared to conventional melt mixing, the milled sample exhibited better melt flowability, flame retardancy, and mechanical strength. Following the same idea, Prabhakar and coworkers reported the fabrication of flame-retardant thermoplastic starch/flax fabric green composites [117]. In this work, the starch was first plasticized into thermoplastic starch, via a ball milling process. The starch, flax fabric (FF), and APP were then mixed, to develop the flame-retardant composites. Pi and coworkers studied the effect of high-energy ball milling on the poly(vinyl chloride) (PVC)/zinc borate (ZB)/aluminum trihydrate (ATH) systems [118]. The first sample was PVC with ZB, and the second was PVC with ZN-ATH. The third sample was PVC with a mixture of ZB and ATH. It was found that high-energy ball milling induced the chemical bonding between PVC and ZB or ZB–ATH. As a result, an enhancement in the LOI and mechanical properties was observed. Moreover, the PVC/ZB and PVC/ZB–ATH composites exhibited better fire retardancy in terms of the suppressed release of aromatic compounds.

**Figure 9:**
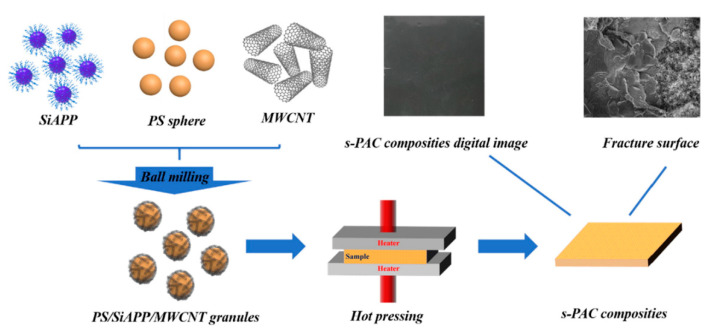
Schematic illustration of the fabrication route of s-PAC composites via ball milling [119]. Reproduced with permission from Elsevier.

In addition to the ball milling-induced mixing, a subsequent hot pressing is always performed to construct a flame-retardant polymer composite with a segregated structure, which is favorable for achieving high electrical conductivity and electromagnetic-wave-shielding performance. Gao and coworkers developed segregated polystyrene (PS) composites with exceptional flame retardancy and electromagnetic wave shielding (EMI) properties, with the assistance of ball milling [119]. As shown in Figure 9, the PS particles, silicon-wrapped ammonium polyphosphate (SiAPP), and MWCNT were ball milled first, to obtain PS/SiAPP/MWCNT granules. It demonstrated that the SiAPP and MWCNT were uniformly distributed onto PS spheres. After hot pressing, a segregated structure s-PAC composite could be obtained, as displayed in the SEM image in Figure 9. Only 7 wt% MWCNT endowed the composites with promising thermal stability and fire retardancy. Specifically, a 60.5% and 33.9% reduction in PHRR and THR, respectively, was reported. Additionally, the EMI shielding property could reach 11 dB. The synergistic effect between MWCNT and SiAPP was believed to be the main contributor to exceptional fire safety, by forming a compact protective char layer on the bottom PS materials. The detailed EMI shielding mechanism was also clarified. Luo and coworkers followed the same strategy, in developing electrically conductive and flame-retardant low-density polyethylene composites with phosphorus-nitrogen-based flame retardant and MWCNTs, using a ball milling and hot-pressing method [120]. Notably, a dramatic decrease in PHRR (49.8%) and THR (51.9%) was observed. Therefore, the combination of ball milling and hot pressing is feasible to construct polymer composites with segregated structures, to achieve multifunctionality, including fire retardancy, thermal and electrical conductivity, and EMI performance. It is an up-and-coming technique for achieving the facile preparation of flame-retardant polymer materials.

## 5. Challenges and Prospects

Although progress has been made in the ball milling-promoted facile preparation of flame-retardant polymer materials, some key points need to be addressed when considering its extensive application. Firstly, limited by the basic operation principle, the products obtained by ball milling are not always homogeneous. Compared to conventional wet-chemistry synthesis, the scalability and consistency of ball milling are always limited [121,122]. To avoid this problem, the ball milling procedures can be varied, including changing the rotation direction appropriately. Sometimes, the impurities from the ball mill tank and beads are nonnegligible, due to the violent collision. Selecting the milling containers and balls with higher hardness is preferable, to reduce the chance of contamination. Furthermore, the ball milling process always lasts for a long time, which is time-consuming, and produces flame retardants in batch mode [123]. To improve efficiency, a processing additive is highly recommended. For example, the intercalators are efficient in assisting the exfoliation of layered compounds using the ball milling method. The configuration of the ball milling machine to achieve the preparation of flame retardants in flow mode is challenging, and of high importance. In addition, ball milling for the solid-state synthesizing of new flame retardants is in its infancy at present, and requires extensive research work to achieve its full potential and industrial value. Moreover, the mixing action of ball milling for a flame-retardant additive and a polymer matrix can be multipurpose, rather than the simple blending of various particles. Using the proper design, some promising polymer composites could be fabricated, such as composites with segregated structures. More customized and specific structures are highly desirable, with the assistance of ball milling.

## 6. Conclusions

In this paper, we review the progress in the development of flame retardants and flame-retardant polymer composites using the ball milling method. Starting from the basic concept and category of ball milling, two types of ball milling machine—planetary ball milling, and high-energy ball milling—are introduced. Due to the generated impact and shear forces, and the high-temperature surroundings, ball milling can achieve the crushing, exfoliating, modification, and chemical reaction required in the preparation of flame retardants. The simultaneous exfoliation and functionalization of layer-compound-based flame retardants are clearly introduced. In addition, the mixing action of the flame retardants and the polymer matrix using the ball milling approach is described, especially the part in which segregated structures are constructed for multifunctional purposes. Flame-retardant polymer composites with segregated structure have exhibited a promising application in electric products, which always require electrical conductivity and an electromagnetic shielding function, while facing high fire risk. Despite the rapid development of ball-milled flame retardants, some challenges and prospective developments are proposed, to promote their practical applications, including heterogeneity issues, impurity problems, and the process being time-consuming. Nevertheless, the authors believe that in the next 10 years, many more flame retardants and flame-retardant polymer composite products prepared by the ball milling method will be coming out of research labs, and will be commercialized across industries.

## Data Availability

Not applicable.

## References

[1] Alexandre M., Dubois P. (2000). Polymer-layered silicate nanocomposites: Preparation, properties and uses of a new class of materials. Mater. Sci. Eng. R Rep..

[2] Stuart M.A.C., Huck W.T.S., Genzer J., Muller M., Ober C., Stamm M., Sukhorukov G.B., Szleifer I., Tsukruk V.V., Urban M. (2010). Emerging applications of stimuli-responsive polymer materials. Nat. Mater..

[3] Qiu H.Y., Feng K., Gapeeva A., Meurisch K., Kaps S., Li X., Yu L.M., Mishra Y.K., Adelung R., Baum M. (2022). Functional polymer materials for modern marine biofouling control. Prog. Polym. Sci..

[4] Fico D., Rizzo D., Casciaro R., Corcione C.E. (2022). A Review of Polymer-Based Materials for Fused Filament Fabrication (FFF): Focus on Sustainability and Recycled Materials. Polymers.

[5] Huang Q.D., Chen J.A., Shao X.C., Zhang L., Dong Y.J., Li W.J., Zhang C., Ma Y.G. (2023). New electropolymerized triphenylamine polymer films and excellent multifunctional electrochromic energy storage system materials with real-time monitoring of energy storage status. Chem. Eng. J..

[6] Oladapo B.I., Kayode J.F., Akinyoola J.O., Ikumapayi O.M. (2023). Shape memory polymer review for flexible artificial intelligence materials of biomedical. Mater. Chem. Phys..

[7] Chen R.S., Ahmad S., Gan S., Salleh M.N., Ab Ghani M.H., Tarawneh M.A. (2016). Effect of polymer blend matrix compatibility and fibre reinforcement content on thermal stability and flammability of ecocomposites made from waste materials. Thermochim. Acta.

[8] Kalali E.N., Zhang L., Shabestari M.E., Croyal J., Wang D.Y. (2019). Flame-retardant wood polymer composites (WPCs) as potential fire safe bio-based materials for building products: Preparation, flammability and mechanical properties. Fire Saf. J..

[9] Mohanty D., Chen S.Y., Hung I.M. (2022). Effect of Lithium Salt Concentration on Materials Characteristics and Electrochemical Performance of Hybrid Inorganic/Polymer Solid Electrolyte for Solid-State Lithium-Ion Batteries. Batteries.

[10] Sahinoz M., Aruntas H.Y., Guru M. (2022). Processing of polymer wood composite material from pine cone and the binder of phenol formaldehyde/PVAc/molasses and improvement of its properties. Case Stud. Constr. Mater..

[11] Vahabi H., Jouyandeh M., Parpaite T., Saeb M.R., Ramakrishna S. (2021). Coffee Wastes as Sustainable Flame Retardants for Polymer Materials. Coatings.

[12] Zhao P., Tian L., Guo Y., Lv B., Mao X., Li T., Cui J., Guo J., Yang B. (2022). A facile method to prepare high-performance thermal insulation and flame retardant materials from amine-linked porous organic polymers. Eur. Polym. J..

[13] Vahabi H., Laoutid F., Formela K., Saeb M.R., Dubois P. (2022). Flame-Retardant Polymer Materials Developed by Reactive Extrusion: Present Status and Future Perspectives. Polym. Rev..

[14] Chen W., Liu P., Liu Y., Liu Z. (2022). Recent advances in Two-dimensional Ti_3_C_2_T_x_ MXene for flame retardant polymer materials. Chem. Eng. J..

[15] Salamova A., Hermanson M.H., Hites R.A. (2014). Organophosphate and Halogenated Flame Retardants in Atmospheric Particles from a European Arctic Site. Environ. Sci. Technol..

[16] Harrad S., Drage D., Sharkey M., Stubbings W., Alghamdi M., Berresheim H., Coggins M., Rosa A.H. (2023). Elevated concentrations of halogenated flame retardants in waste childcare articles from Ireland. Environ. Pollut..

[17] Ren H.L., Ge X., Qi Z.H., Lin Q.H., Shen G.F., Yu Y.X., An T.C. (2023). Emission and gas-particle partitioning characteristics of atmospheric halogenated and organophosphorus flame retardants in decabromodiphenyl ethane-manufacturing functional areas. Environ. Pollut..

[18] Kerric A., Mazerolle M.J., Giroux J.F., Verreault J. (2023). Halogenated flame retardant exposure pathways in urban-adapted gulls: Are atmospheric routes underestimated?. Sci. Total Environ..

[19] Capozzi S.L., Lehman D.C., Venier M. (2023). Disentangling Source Profiles and Time Trends of Halogenated Flame Retardants in the Great Lakes. Environ. Sci. Technol..

[20] Eo S.-M., Cha E., Kim D.-W. (2009). Effect of an inorganic additive on the cycling performances of lithium-ion polymer cells assembled with polymer-coated separators. J. Power Sources.

[21] Katase F., Kajiyama S., Kato T. (2017). Macromolecular templates for biomineralization-inspired crystallization of oriented layered zinc hydroxides. Polym. J..

[22] Sangian D., Ide Y., Bando Y., Rowan A.E., Yamauchi Y. (2018). Materials Nanoarchitectonics Using 2D Layered Materials: Recent Developments in the Intercalation Process. Small.

[23] Han X., Li N., Wu B., Li D., Pan Q., Wang R. (2022). Microstructural characterization and corrosion resistance evaluation of chromate-phosphate/water-soluble resin composite conversion coating on Al surfaces. Prog. Org. Coat..

[24] Gao F., Tong L.F., Fang Z.P. (2006). Effect of a novel phosphorous-nitrogen containing intumescent flame retardant on the fire retardancy and the thermal behaviour of poly(butylene terephthalate). Polym. Degrad. Stab..

[25] Wang D.-L., Liu Y., Wang D.-Y., Zhao C.-X., Mou Y.-R., Wang Y.-Z. (2007). A novel intumescent flame-retardant system containing metal chelates for polyvinyl alcohol. Polym. Degrad. Stab..

[26] Xue M., Zhang X., Wu Z., Wang H., Gu Z., Bao C., Tian X. (2014). A Commercial Phosphorous-Nitrogen Containing Intumescent Flame Retardant for Thermoplastic Polyurethane. J. Appl. Polym. Sci..

[27] Wang C., Wu Y., Li Y., Shao Q., Yan X., Han C., Wang Z., Liu Z., Guo Z. (2018). Flame-retardant rigid polyurethane foam with a phosphorus-nitrogen single intumescent flame retardant. Polym. Adv. Technol..

[28] Anilkumar Y., Felipe M., de Souza T.D., Ram K. (2022). Gupta, Recent Advancements in Flame-Retardant Polyurethane Foams: A Review. Ind. Eng. Chem. Res..

[29] Jiang Y., Yang H., Lin X., Xiang S., Feng X., Wan C. (2023). Surface Flame-Retardant Systems of Rigid Polyurethane Foams: An Overview. Materials.

[30] Li F. (2023). Comprehensive Review of Recent Research Advances on Flame-Retardant Coatings for Building Materials: Chemical Ingredients, Micromorphology, and Processing Techniques. Molecules.

[31] Palacios E., Leret P., De La Mata M.J., Fernandez J.F., De Aza A.H., Rodriguez M.A., Rubio-Marcos F. (2016). Self-Forming 3D Core-Shell Ceramic Nanostructures for Halogen-Free Flame Retardant Materials. ACS Appl. Mater. Interfaces.

[32] Xue B., Niu M., Yang Y., Bai J., Song Y., Peng Y., Liu X. (2018). Multi-functional carbon microspheres with double shell layers for flame retardant poly (ethylene terephthalate). Appl. Surf. Sci..

[33] Holdsworth A.F., Horrocks A.R., Kandola B.K. (2020). Novel metal complexes as potential synergists with phosphorus based flame retardants in polyamide 6.6. Polym. Degrad. Stab..

[34] Holdsworth A.F., Horrocks A.R., Kandola B.K. (2020). Potential Synergism between Novel Metal Complexes and Polymeric Brominated Flame Retardants in Polyamide 6.6. Polymers.

[35] Horrocks A.R. (2020). The Potential for Bio-Sustainable Organobromine-Containing Flame Retardant Formulations for Textile Applications—A Review. Polymers.

[36] Do J.-L., Friščić T. (2017). Mechanochemistry: A Force of Synthesis. ACS Cent. Sci..

[37] Burmeister C.F., Kwade A. (2013). Process engineering with planetary ball mills. Chem. Soc. Rev..

[38] Yan H., Dong B., Du X., Ma S., Wei L., Xu B. (2014). Flame-Retardant Performance of Polystyrene Enhanced by Polyphenylene Oxide and Intumescent Flame Retardant. Polym. Plast. Technol. Eng..

[39] Zhang T., Kuga S., Wu M., Huang Y. (2020). Chitin Nanofibril-Based Flame Retardant for Paper Application. ACS Sustain. Chem. Eng..

[40] Hwang S., Grätz S., Borchardt L. (2022). A guide to direct mechanocatalysis. Chem. Commun..

[41] Zhang Q., Saito F. (2012). A review on mechanochemical syntheses of functional materials. Adv. Powder Technol..

[42] Blumbergs E., Serga V., Shishkin A., Goljandin D., Shishko A., Zemcenkovs V., Markus K., Baronins J., Pankratov V. (2022). Selective Disintegration-Milling to Obtain Metal-Rich Particle Fractions from E-Waste. Metals.

[43] Tsuzuki T., McCormick P.G. (2004). Mechanochemical synthesis of nanoparticles. J. Mater. Sci..

[44] Nikolic N., Marinkovic Z., Sreckovic T. (2004). The influence of grinding conditions on the mechanochemical synthesis of zinc stannate. J. Mater. Sci..

[45] Palazon F., Ajjouri Y.E., Sebastia-Luna P., Lauciello S., Manna L., Bolink H.J. (2019). Mechanochemical synthesis of inorganic halide perovskites: Evolution of phase-purity, morphology, and photoluminescence. J. Mater. Chem. C.

[46] Kim M.-J., Jeon I.-Y., Seo J.-M., Dai L., Baek J.-B. (2014). Graphene Phosphonic Acid as an Efficient Flame Retardant. ACS Nano.

[47] Jeon I.-Y., Shin S.-H., Choi H.-J., Yu S.-Y., Jung S.-M., Baek J.-B. (2017). Heavily aluminated graphene nanoplatelets as an efficient flame-retardant. Carbon.

[48] Chen Y., Wu H., Duan R., Zhang K., Meng W., Li Y., Qu H. (2022). Graphene doped Sn flame retardant prepared by ball milling and synergistic with hexaphenoxy cyclotriphosphazene for epoxy resin. J. Mater. Res. Technol..

[49] Qiu S., Hou Y., Xing W., Ma C., Zhou X., Liu L., Kan Y., Yuen R.K.K., Hu Y. (2018). Self-assembled supermolecular aggregate supported on boron nitride nanoplatelets for flame retardant and friction application. Chem. Eng. J..

[50] Chen S., Xu R., Liu J., Zou X., Qiu L., Kang F., Liu B., Cheng H.-M. (2019). Simultaneous Production and Functionalization of Boron Nitride Nanosheets by Sugar-Assisted Mechanochemical Exfoliation. Adv. Mater..

[51] Han G., Zhao X., Feng Y., Ma J., Zhou K., Shi Y., Liu C., Xie X. (2021). Highly flame-retardant epoxy-based thermal conductive composites with functionalized boron nitride nanosheets exfoliated by one-step ball milling. Chem. Eng. J..

[52] Han G., Zhang D., Kong C., Zhou B., Shi Y., Feng Y., Liu C., Wang D.-Y. (2022). Flexible, thermostable and flame-resistant epoxy-based thermally conductive layered films with aligned ionic liquid-wrapped boron nitride nanosheets via cyclic layer-by-layer blade-casting. Chem. Eng. J..

[53] Guo J., Yang L., Zhang L., Li C. (2022). Simultaneous exfoliation and functionalization of black phosphorus by sucrose-assisted ball milling with NMP intercalating and preparation of flame retardant polyvinyl alcohol film. Polymer.

[54] Duan Z., Wang Y., Bian S., Liu D., Zhang Y., Zhang X., He R., Wang J., Qu G., Chu P.K. (2022). Size-dependent flame retardancy of black phosphorus nanosheets. Nanoscale.

[55] He L., Wang J., Wang B., Wang X., Zhou X., Cai W., Mu X., Hou Y., Hu Y., Song L. (2019). Large-scale production of simultaneously exfoliated and Functionalized MXenes as promising flame retardant for polyurethane. Compos. B. Eng..

[56] Chen X., Zhao Z., Hao M., Wang D. (2013). Research of hydrogen generation by the reaction of Al-based materials with water. J. Power Sources.

[57] Xie L., Ding Y., Ren J., Xie T., Qin Y., Wang X., Chen F. (2021). Improved Hydrogen Generation Performance via Hydrolysis of MgH_2_ with Nb_2_O_5_ and CeO_2_ Doping. Mater. Trans..

[58] Wang R., Zhu Z.X., Tan S.F., Guo J., Xu Z.M. (2020). Mechanochemical degradation of brominated flame retardants in waste printed circuit boards by Ball Milling. J. Hazard. Mater..

[59] Guo X., Geng J., Sun B., Xu Q., Li Y., Xie S., Xue Y., Yan H. (2021). Great enhancement of efficiency of intumescent flame retardants by titanate coupling agent and polysiloxane. Polym. Adv. Technol..

[60] Lei Y., Bai Y., Shi Y., Liang M., Zou H., Zhou S. (2022). Composite nanoarchitectonics of poly(vinylidene fluoride)/graphene for thermal and electrical conductivity enhancement via constructing segregated network structure. J. Polym. Res..

[61] Antonio Puertolas J., Jose Martinez-Morlanes M., Javier Pascual F., Morimoto T. (2023). Influence of mechanical blending method and consolidation temperature on electrical properties of the prepared graphene nanoplatelet/UHMWPE composite. J. Polym. Res..

[62] Kumar M., Xiong X., Wan Z., Sun Y., Tsang D.C.W., Gupta J., Gao B., Cao X., Tang J., Ok Y.S. (2020). Ball milling as a mechanochemical technology for fabrication of novel biochar nanomaterials. Bioresour. Technol..

[63] Dudina D.V., Bokhonov B.B. (2022). Materials Development Using High-Energy Ball Milling: A Review Dedicated to the Memory of M.A. Korchagin. J. Compos. Sci..

[64] Bocz K., Krain T., Marosi G. (2015). Effect of Particle Size of Additives on the Flammability and Mechanical Properties of Intumescent Flame Retarded Polypropylene Compounds. Int. J. Polym. Sci..

[65] Bao Y., Li X., Tang P., Liu C., Zhang W., Ma J. (2019). Attapulgite modified cotton fabric and its flame retardancy. Cellulose.

[66] Üreyen M.E., Kaynak E. (2019). Effect of Zinc Borate on Flammability of PET Woven Fabrics. Adv. Polym. Technol..

[67] Jawaid M., Kian L.K., Alamery S., Saba N., Fouad H., Alothman O.Y., Sain M. (2022). Development and characterization of fire retardant nanofiller from date palm biomass. Biomass Convers. Bior..

[68] Azizi H., Ahmad F., Yusoff P.S.M.M., Zia-ul-Mustafa M.I. Fire Performance, Microstructure and Thermal Degradation of an Epoxy Based Nano Intumescent Fire Retardant Coating for Structural Applications. Proceedings of the 23rd Scientific Conference of Microscopy-Society-Malaysia (SCMSM), Univ Teknologi Petronas.

[69] Andrikopoulos K.S., Bounos G., Lainioti G.C., Ioannides T., Kallitsis J.K., Voyiatzis G.A. (2022). Flame Retardant Nano-Structured Fillers from Huntite/Hydromagnesite Minerals. Nanomaterials.

[70] Pate R., Klise G., Wu B. (2011). Resource demand implications for US algae biofuels production scale-up. Appl. Energy.

[71] Siebert H.M., Wilker J.J. (2019). Deriving Commercial Level Adhesive Performance from a Bio-Based Mussel Mimetic Polymer. ACS Sustain. Chem. Eng..

[72] Gupta I., Gupta O. (2023). Recent Advancements in the Recovery and Reuse of Organic Solvents Using Novel Nanomaterial-Based Membranes for Renewable Energy Applications. Membranes.

[73] Wang H., Zhu Z., Yuan J., Wang H., Wang Z., Yang F., Zhan J., Wang L. (2021). A new recycling strategy for preparing flame retardants from polyphenylene sulfide waste textiles. Compos. Commun..

[74] Dragoman M., Dragoman D. (2009). Graphene-based quantum electronics. Prog. Quantum Electron..

[75] Wang H., Wang H.S., Ma C., Chen L., Jiang C., Chen C., Xie X., Li A.-P., Wang X. (2021). Graphene nanoribbons for quantum electronics. Nat. Rev. Phys..

[76] Julkapli N.M., Bagheri S. (2015). Graphene supported heterogeneous catalysts: An overview. Int. J. Hydrogen Energy.

[77] Alaf M., Tocoglu U., Kartal M., Akbulut H. (2016). Graphene supported heterogeneous catalysts for LiO_2_ batteries. Appl. Surf. Sci..

[78] Ratnikov P.V., Silin A.P. (2008). Planar Graphene-Narrow-Gap Semiconductor-Graphene Heterostructure. Bull. Lebedev Phys. Inst..

[79] Ebrahimi M., Horri A., Sanaeepur M., Tavakoli M.B. (2020). Tight-binding description of graphene-BCN-graphene layered semiconductors. J. Comput. Electron..

[80] Zabihi O., Ahmadi M., Li Q., Ferdowsi M.R.G., Mahmoodi R., Kalali E.N., Wang D.-Y., Naebe M. (2020). A sustainable approach to scalable production of a graphene based flame retardant using waste fish deoxyribonucleic acid. J. Clean. Prod..

[81] Yang P.X., Wu H.G., Yang F.F., Yang J., Wang R., Zhu Z.G. (2021). A Novel Self-Assembled Graphene-Based Flame Retardant: Synthesis and Flame Retardant Performance in PLA. Polymers.

[82] Wang C., Wang J., Men Z., Wang Y., Han Z. (2019). Thermal Degradation and Combustion Behaviors of Polyethylene/Alumina Trihydrate/Graphene Nanoplatelets. Polymers.

[83] Tran V.Q., Doan H.T., Nguyen N.T., Do C.V. (2019). Preparation of Graphene Nanoplatelets by Thermal Shock Combined with Ball Milling Methods for Fabricating Flame-Retardant Polymers. J. Chem..

[84] Duan R., Wu H., Li J., Zhou Z., Meng W., Liu L., Qu H., Xu J. (2022). Phosphor nitrile functionalized UiO-66-NH_2_/graphene hybrid flame retardants for fire safety of epoxy. Colloids Surf. A.

[85] Li J., Lyu Y., Li C., Zhang F., Li K., Li X., Li J., Kim K.-H. (2023). Development of strong, tough and flame-retardant phenolic resins by using Acacia mangium tannin-functionalized graphene nanoplatelets. Int. J. Biol. Macromol..

[86] Xu X., Jiang Z., Zhu K., Zhang Y., Zhu M., Wang C., Wang H., Ren A. (2022). Highly flame-retardant and low toxic polybutylene succinate composites with functionalized BN@APP exfoliated by ball milling. J. Appl. Polym. Sci..

[87] Gibertini E., Carosio F., Aykanat K., Accogli A., Panzeri G., Magagnin L. (2021). Silica-encapsulated red phosphorus for flame retardant treatment on textile. Surf. Interfaces.

[88] Chen X., Lan W., Dou W. (2022). Polystyrene nanospheres coated red phosphorus flame retardant for polyamide 66. J. Appl. Polym. Sci..

[89] Yin S., Ren X., Lian P., Zhu Y., Mei Y. (2020). Synergistic Effects of Black Phosphorus/Boron Nitride Nanosheets on Enhancing the Flame-Retardant Properties of Waterborne Polyurethane and Its Flame-Retardant Mechanism. Polymers.

[90] Qiu S., Yang W., Wang X., Hu Y. (2023). Phthalocyanine zirconium diazo passivation of black phosphorus for efficient smoke suppression, flame retardant and mechanical enhancement. Chem. Eng. J..

[91] Qu Z., Wu K., Xu C.-A., Li Y., Jiao E., Chen B., Meng H., Cui X., Wang K., Shi J. (2021). Facile Construction of a Flexible Film with Ultrahigh Thermal Conductivity and Excellent Flame Retardancy for a Smart Fire Alarm. Chem. Mater..

[92] Qu Z., Wang K., Xu C.-A., Li Y., Jiao E., Chen B., Meng H., Cui X., Shi J., Wu K. (2021). Simultaneous enhancement in thermal conductivity and flame retardancy of flexible film by introducing covalent bond connection. Chem. Eng. J..

[93] Zou B., Qiu S., Qian Z., Wang J., Zhou Y., Xu Z., Yang W., Xing W. (2021). Phosphorus/Nitrogen-Codoped Molybdenum Disulfide/Cobalt Borate Nanostructures for Flame-Retardant and Tribological Applications. ACS Appl. Nano Mater..

[94] Yang Z., Kang X., Lu S., Wang Z., Fang X., Li J., Liu B., Ding T., Xu Y. (2023). Synergistic effects of molybdenum disulfide on a novel intumescent flame retardant polyformaldehyde system. J. Appl. Polym. Sci..

[95] Qiu S., Hu Y., Shi Y., Hou Y., Kan Y., Chu F., Sheng H., Yuen R.K.K., Xing W. (2018). In situ growth of polyphosphazene particles on molybdenum disulfide nanosheets for flame retardant and friction application. Compos. Part A Appl..

[96] Wang X., Ji H., Wang F., Cui X., Liu Y., Du X., Lu X. (2021). NiFe_2_O_4_-based magnetic covalent organic framework nanocomposites for the efficient adsorption of brominated flame retardants from water. Microchim. Acta.

[97] Peng H., Mao Y., Wang D., Fu S. (2022). B-N-P-linked covalent organic frameworks for efficient flame retarding and toxic smoke suppression of polyacrylonitrile composite fiber. Chem. Eng. J..

[98] Mu X., Zhan J., Feng X., Yuan B., Qiu S., Song L., Hu Y. (2017). Novel Melamine/o-Phthalaldehyde Covalent Organic Frameworks Nanosheets: Enhancement Flame Retardant and Mechanical Performances of Thermoplastic Polyurethanes. ACS Appl. Mater. Interfaces.

[99] Mu X., Pan Y., Ma C., Zhan J., Song L. (2018). Novel Co_3_O_4_/covalent organic frameworks nanohybrids for conferring enhanced flame retardancy, smoke and CO suppression and thermal stability to polypropylene. Mater. Chem. Phys..

[100] Ren J., Wang Y., Piao J., Cui J., Guan H., Jiao C., Chen X. (2023). Facile construction of phosphorus-free and green organic-inorganic hybrid flame-retardant system: For improving fire safety of EP. Prog. Org. Coat..

[101] Ren J., Wang Y., Piao J., Ou M., Lian R., Cui J., Guan H., Liu L., Jiao C., Chen X. (2023). Facile construction of organic–inorganic hybrid flame-retardant system based on fully biomass: Improving the fire safety and mechanical property of epoxy resin. Chem. Eng. J..

[102] Ren J., Piao J., Wang Y., Wang Y., Feng T., Liu L., Jiao C., Chen X. (2023). Facile synthesis of bio-based phosphorus/nitrogen compound for high efficiency flame retardant finishing of cotton fabric. Cellulose.

[103] Huang S.-C., Deng C., Chen H., Li Y.-M., Zhao Z.-Y., Wang S.-X., Wang Y.-Z. (2019). Novel Ultrathin Layered Double Hydroxide Nanosheets with In Situ Formed Oxidized Phosphorus as Anions for Simultaneous Fire Resistance and Mechanical Enhancement of Thermoplastic Polyurethane. ACS Appl. Polym. Mater..

[104] Bi X., Meng W., Meng Y., Di H., Li J., Xie J., Xu J., Fang L. (2021). Novel [BMIM]PF_6_ modified flake-ANP flame retardant: Synthesis and application in epoxy resin. Polym. Test..

[105] Ou H., Xu J., Liu B., Xue H., Weng Y., Jiang J., Xu G. (2021). Study on synergistic expansion and flame retardancy of modified kaolin to low density polyethylene. Polymer.

[106] Wang Y., Piao J., Ren J., Feng T., Wang Y., Liu W., Dong H., Chen W., Jiao C., Chen X. (2023). Simultaneously improving the hydrophobic property and flame retardancy of aluminum hypophosphite using rare earth based coupling agent for epoxy composites. Polym. Adv. Technol..

[107] Członka S., Kairytė A., Miedzińska K., Strąkowska A. (2021). Polyurethane Hybrid Composites Reinforced with Lavender Residue Functionalized with Kaolinite and Hydroxyapatite. Materials.

[108] Miedzińska K., Członka S., Strąkowska A., Strzelec K. (2021). Vermiculite Filler Modified with Casein, Chitosan, and Potato Protein as a Flame Retardant for Polyurethane Foams. Int. J. Polym. Sci..

[109] Członka S., Kairytė A., Miedzińska K., Strąkowska A. (2021). Polyurethane Composites Reinforced with Walnut Shell Filler Treated with Perlite, Montmorillonite and Halloysite. Int. J. Mol. Sci..

[110] Niu L., Xu J., Yang W., Zhao J., Su J., Guo Y., Liu X. (2018). Research on nano-Sb_2_O_3_ flame retardant in char formation of PBT. Ferroelectrics.

[111] Díez-Pascual A.M., Xu C., Luque R. (2014). Development and characterization of novel poly(ether ether ketone)/ZnO bionanocomposites. J. Mater. Chem. B.

[112] Chen Z., Guo Y., Chu Y., Chen T., Zhang Q., Li C., Jiang J., Chen T., Yu Y., Liu L. (2022). Solvent-free and electron transfer-induced phosphorus and nitrogen-containing heterostructures for multifunctional epoxy resin. Compos. B Eng..

[113] Fiss B.G., Hatherly L., Stein R.S., Friščić T., Moores A. (2019). Mechanochemical Phosphorylation of Polymers and Synthesis of Flame-Retardant Cellulose Nanocrystals. ACS Sustain. Chem. Eng..

[114] Zhang T., Wu M., Kuga S., Ewulonu C.M., Huang Y. (2020). Cellulose Nanofibril-Based Flame Retardant and Its Application to Paper. ACS Sustain. Chem. Eng..

[115] Xu J., Liu X., Yang W., Niu L., Zhao J., Ma B., Kang C. (2019). Influence of montmorillonite on the properties of halogen–antimony flame retardant polypropylene composites. Polym. Compos..

[116] Liu Y., Li J., Wang Q. (2007). Preparation of High Loading Magnesium Hydroxide Flame Retardant Polypropylene by Solid State Shear Milling. J. Compos. Mater..

[117] Prabhakar M.N., Rehman Shah A.U., Song J.-I. (2017). Improved flame-retardant and tensile properties of thermoplastic starch/flax fabric green composites. Carbohydr. Polym..

[118] Pi H., Guo S., Ning Y. (2003). Mechanochemical improvement of the flame-retardant and mechanical properties of zinc borate and zinc borate-aluminum trihydrate-filled poly(vinyl chloride). J. Appl. Polym. Sci..

[119] Gao C., Shi Y., Chen Y., Zhu S., Feng Y., Lv Y., Yang F., Liu M., Shui W. (2022). Constructing segregated polystyrene composites for excellent fire resistance and electromagnetic wave shielding. J. Colloid Interface Sci..

[120] Luo Y., Xie Y., Chen R., Zheng R., Wu H., Sheng X., Xie D., Mei Y. (2021). A low-density polyethylene composite with phosphorus-nitrogen based flame retardant and multi-walled carbon nanotubes for enhanced electrical conductivity and acceptable flame retardancy. Front. Chem. Sci. Eng..

[121] Mio H., Kano J., Saito F. (2004). Scale-up method of planetary ball mill. Chem. Eng. Sci..

[122] Santhanam P.R., Dreizin E.L. (2012). Predicting conditions for scaled-up manufacturing of materials prepared by ball milling. Powder Technol..

[123] Holdsworth A.F., Eccles H., Halman A.M., Mao R., Bond G. (2018). Low-Temperature Continuous Flow Synthesis of Metal Ammonium Phosphates. Sci. Rep..

